# ATP6V1B1 regulates ovarian cancer progression and cisplatin sensitivity through the mTOR/autophagy pathway

**DOI:** 10.1007/s11010-024-05025-w

**Published:** 2024-05-12

**Authors:** Shien Mo, Tingji Liu, Haiqin Zhou, Junning Huang, Ling Zhao, Fangfang Lu, Yan Kuang

**Affiliations:** 1 Department of Gynecology, First Affiliated Hospital of Guangxi Medical University, Nanning, Guangxi China; 2 Key Laboratory of Early Prevention and Treatment for Regional High Frequency Tumor, Gaungxi Medical University, Ministry of Education, Nanning, Guangxi China; 3 Guangxi Key Laboratory of High-Incidence-Tumor Prevention & Treatment, Guangxi Medical University, Nanning, Guangxi China

**Keywords:** V-ATPase, ATP6V1B1, mTOR, Autophagy, Cisplatin sensitivity, Ovarian cancer

## Abstract

Early detection and effective chemotherapy for ovarian cancer, a serious gynecological malignancy, require further progress. This study aimed to investigate the molecular mechanism of ATPase H^+^-Transporting V1 Subunit B1 (ATP6V1B1) in ovarian cancer development and chemoresistance. Our data show that ATP6V1B1 is upregulated in ovarian cancer and correlated with decreased progression-free survival. Gain- and loss-of-function experiments demonstrated that ATP6V1B1 promotes the proliferation, migration, and invasion of ovarian cancer cells in vitro, while ATP6V1B1 knockout inhibits tumor growth in vivo. In addition, knocking down ATP6V1B1 increases the sensitivity of ovarian cancer cells to cisplatin. Mechanistic studies showed that ATP6V1B1 regulates the activation of the mTOR/autophagy pathway. Overall, our study confirmed the oncogenic role of ATP6V1B1 in ovarian cancer and revealed that ATP6V1B1 promotes ovarian cancer progression via the mTOR/autophagy axis.

**Supplementary Information:**

The online version contains supplementary material available at 10.1007/s11010-024-05025-w.

## Introduction

Ovarian cancer is one of the most prevalent malignant tumors in gynecology. It is often difficult to detect in its early stages due to the lack of effective screening methods, leading to a high mortality rate. Ovarian cancer is the second leading cause of mortality among gynecological tumors, followed by cervical cancer. Epithelial ovarian cancer accounts for more than 70% of all malignant ovarian tumors. Despite continuous health care development, which has significantly improved the 5-year survival rate for many cancer patients, it is regrettable that, in comparison to the nearly 85% 5-year survival rate for breast cancer patients, the survival rate for ovarian cancer patients has barely improved, even in developed countries [1]. The primary treatment for ovarian cancer is surgical removal of the tumor, platinum-based chemotherapy, and targeted therapies. The objective of chemotherapy is to induce DNA damage and the subsequent death of cancer cells. However, many patients experience a relapse due to chemotherapy resistance [2]. Targeted agents, such as bevacizumab and PARP inhibitors, have emerged as potential first-line or maintenance therapies [3]. Despite considerable research efforts, the overall survival rate has not shown marked improvement. Therefore, innovative molecular markers and insights into platinum resistance remain crucial areas of research in the field of ovarian cancer.

The vacuolar-type H^+^-ATPase (V-ATPase) is a transporter protein that is found in eukaryotic organelles, such as vacuoles, lysosomes, and endosomes. V-ATPase is involved in various physiological processes of the cell by regulating proton transport and signal transduction. Its role in acidifying intracellular compartments and regulating signal transduction makes it an important player in maintaining normal cell function [4]. Recently, the association of V-ATPase with cancer has garnered substantial attention [5]. High expression of V-ATPase has been associated with poor survival outcomes in patients with breast cancer, ovarian cancer, pancreatic cancer and esophageal cancer [6–10]. Tumor cells exhibit a high expression of V-ATPase, which maintains an internal alkaline and external acidic environment. An alkaline intracellular environment is conducive to tumor cell proliferation, whereas an acidic extracellular environment facilitates tumor cell invasion and migration by enhancing protease and matrix metalloproteinase (MMP) activity [11, 12]. ATPase H^+^ Transporting V1 Subunit B1 (ATP6V1B1), also known as VATB, ATP6B1, RTA1B, VPP3, and V-ATPase Subunit B1, is one of the three B subunits of the V1 domain of V-ATPase. A study conducted by Kulshrestha et al. [13] demonstrated that the ATPase-V0a2 enzyme is overexpressed in ovarian cancer cells and has the capacity to enhance cellular resistance to cisplatin. However, reports on the role of the V-ATPase V1 domain and its subunits in ovarian cancer are scarce.

Autophagy is a cellular process whereby cells form a bilayer membrane to remove damaged organelles, misfolded proteins and other cytoplasmic components [14]. As a form of nonprogrammed cell death, autophagy plays a multifaceted role in cancer; protective autophagy supports tumor growth [15, 16], while lethal autophagy encourages tumor cell apoptosis [17, 18]. Apoptosis is a type of programmed cell death that directs cells to commit suicide in response to external and internal stimuli [19]. Inducing apoptosis in tumor cells or activating cell death-related pathways is one of the strategies to inhibit the growth of tumor cells. The Bcl-2 protein, which functions as a coregulator of apoptosis and autophagy, has been demonstrated to link these two processes. The ATPase enzyme is known to regulate autophagy by altering the lysosomal environment, or by activating the mTOR pathway [20].

A recent study has revealed that ATP6V1B1 is overexpressed in ovarian cancer tissues and platinum-resistant ovarian tissues. The role of ATP6V1B1 in regulating the cell cycle is well established, but there is a dearth of information regarding the mechanisms by which ATP6V1B1 affects cell death and platinum resistance [21]. The objective of this study was to investigate the potential role of ATP6V1B1 in the progression of ovarian cancer and its ability to regulate the mechanism of ovarian cancer cell death, to identify insights and therapeutic targets for the diagnosis and treatment of ovarian cancer.

## Materials and methods

### Tissue specimens

Ovarian tissue samples and paraffin-embedded specimens were obtained from patients who underwent surgery at the Department of Obstetrics and Gynecology, First Affiliated Hospital of Guangxi Medical University, between 2019 and 2023. All patients have signed a written informed consent form and none of them received any chemotherapy, radiotherapy or targeted therapy before surgery. Normal ovarian specimens were obtained from patients who had uterine myoma, adenomyosis or uterine prolapse. Twenty-four ovarian tissue samples were utilized for PCR analysis. Thirty other paraffin-embedded pathological specimens were sectioned. All tissues were examined and diagnosed by experienced pathologists from the Department of Pathology, First Affiliated Hospital of Guangxi Medical University. This study was approved by the Ethical Review Committee of the First Affiliated Hospital of Guangxi Medical University.

### Cell culture

The normal ovarian epithelial cell line IOSE80 and the ovarian cancer cell lines SKOV-3, A2780 and CAOV3 were purchased from the China Center for Type Culture Collection (CCTCC, Wuhan, China). The cells were cultured in RPMI-1640 medium, except for CAOV3 cells which were cultured in RPMI-DMEM. Both culture media were enriched with 10% fetal bovine serum. All cells were cultured in an atmosphere containing 5% CO_2_ at 37 °C. Once the cells reached approximately 70–80% confluency, they were prepared and plated for experimental purposes.

### Cell transfection and grouping

Plasmid vectors expressing small hairpin RNA (shRNA) targeting ATP6V1B1 and ATP6V1B1 overexpression vectors were constructed (Miaolingbio, Wuhan, China). Cells in a healthy growth state were harvested and seeded into 6-well plates, at the following concentrations per well: SKOV3 (5 × 10^5^) and A2780 (1 × 10^6^). After incubation at 37 °C in a 5% CO_2_ incubator for 16 h, the cells were washed with PBS and then transfected with the aforementioned plasmids using Lipofectamine 3000 (Invitrogen, Carlsbad, California, USA), following the manufacturer’s guidelines.

### RNA extraction and quantitative PCR

RNA was extracted from both the ovarian tissues and the cultured ovarian cancer cells utilizing a Total RNA Extraction Kit (Axygen, USA). Reverse transcription was then performed to synthesize cDNA using the TaKaRa cDNA Synthesis Kit (TaKaRa, Kusatsu, Japan). Quantitative real-time PCR (RT-qPCR) was subsequently conducted with TB Green Premix Ex TaqII (TaKaRa, Kusatsu, Japan) on a CFX Touch Real-Time PCR Machine (Bio-Rad, USA). PCR amplification was performed under the following thermal cycling conditions: initial denaturation at 95 °C for 30 s, followed by 40 cycles of 95 °C for 5 s and 60 °C for 30 s. The sequences of primer used were as follows: ATP6V1B1: F: 5′-CCATGGAGATAGACAGCAGGC-3′, R: 5′-TGGATGGTGGTGTTGTTGC-3′; β-Actin: F: 5′-CCAACCGCGAGAAGATGACC-3′; R: 5′-GAGTCCATCACGATGCCAGT-3′. The expression level of ATP6V1B1 was quantified relative to that of β-actin, which served as the internal control for normalization.

### Cell proliferation and IC_50_

Cells exhibiting optimal growth conditions were seeded into a 96-well cell culture plate at specific seeding densities of 2 × 10^3^ cells per well for SKOV3 cells and 1 × 10^4^ cells per well for A2780 cells. The cells were incubated for 0, 24, 48, 72, or 96 h. Then 100 μL of serum-free 1640 medium containing 10% CCK-8 solution was added to each well. The absorbance of the cells at 450 nm was measured using a Synergy H1 modular multimode microplate reader (BioTek, USA), and the growth curve was plotted based on the optical density (OD) values.

The steps used to determine the IC_50_ of cisplatin in cells were the same as those described above, except that after the cells were incubated in the plate for 24 h, a series of different concentrations of cisplatin were added. The OD values were then measured after 24 h of cisplatin treatment.

For the colony formation assay, cells were seeded into one well of a 6-well plate to evaluate the influence of ATP6V1B1 on cell clonogenicity. To allow colony formation, the cells were cultured for 14 days. The cell colonies were then fixed with 4% paraformaldehyde for 30 min and stained with 0.5% crystal violet for 15 min. The number of colonies was quantified through counting.

### Cell migration and invasion

Cells were seeded into plates and cultured for 24 h in an incubator. Then a single wound was created using a 10 μL pipette tip. Microscopy images were taken to record the distances between cells at 0, 24, or 48 h. For transwell assays, cells suspended in serum-free medium were placed in the upper chambers of transwell inserts (Biofil, Guangzhou, China). The lower chamber was filled with 600 μL of complete medium containing 10% FBS. After 24 h, noninvading cells were removed with cotton swabs. Invading cells were fixed with 4% paraformaldehyde for 25 min and further stained with 0.1% crystal violet for 15 min. The invasion experiment was similar to the migration experiment, except that Matrigel (BD Biosciences) was added to the upper chamber.

### Western blot analysis

Cells and tissues were lysed in RIPA buffer supplemented with 1% PMSF (Solarbio, Beijing, China). Protein concentrations were quantified with a BCA Protein Assay Kit (Beyotime, Shanghai, China). The proteins were separated by SDS‒polyacrylamide gel electrophoresis, and the separated proteins were then transferred to a polyvinylidene difluoride (PVDF) membrane (Millipore, Boston, MA, USA). Then, the membranes were incubated with the following primary antibodies overnight at 4 °C: ATP6V1B1 (1:1000, Proteintech, Wuhan, China), β-actin (1:1000, Proteintech, Wuhan, China), GAPDH (1:10,000, Proteintech, Wuhan, China), mTOR/p-mTOR (1:1000, Abmart, Shanghai, China), P62 (1:2000, Abmart, Shanghai, China), LC3B (1:2000, Abcam, Shanghai, China), Bax (1:1000, Abmart, Shanghai, China), Bcl2 (1:1000, Abmart, Shanghai, China), Caspase-3 (1:1000, Abmart, Shanghai, China), and Cleaved Caspase-3 (1:1000, Abmart, Shanghai, China). The following day, the membranes were incubated with an IgG secondary antibody (1:10,000, Invitrogen, USA) at room temperature. Signal visualization was achieved using an Odyssey CLx (LI-COR, USA).

### Immunohistochemistry

The paraffin sections were deparaffinized in xylene and dehydrated in alcohol, followed by antigen retrieval in sodium citrate buffer. An UltraSensitive™ SP (mouse or rabbit) IHC Kit (Maixin, Fuzhou, China) was used to block endogenous peroxidase activity and the nonspecific staining of the sections. The tissue sections were incubated at 4 °C overnight with the primary antibody, followed by incubation with the biotin labeled secondary antibody the following day. The staining procedure was conducted using a 3,3′-diaminobenzidine (DAB) kit (Maixin, Fuzhou, China), and the resulting sections were photographed using a microscope.

### Apoptosis assay

Cell apoptosis was analyzed using an Annexin V/propidium iodide (PI) apoptosis detection kit (Multi Science, China) in accordance with the manufacturer’s instructions. The cells were incubated in cisplatin for 24 h, followed by staining with Annexin V-APC and AAD. Flow cytometry data were acquired using a BD Accuri C6 Plus and analyzed with FlowJo 10.

### Transmission electron microscopy

The cells were harvested and fixed with prechilled 2.5% glutaraldehyde fixative (Servicebio, Wuhan, China) for 24 h at 4 °C. Subsequently, they were postfixed in 1% osmium tetroxide. After postfixation, the cells were sequentially dehydrated in graded acetone. The cells were then infiltrated with Epon 812 resin and embedded. Ultrathin sections were cut with a diamond knife and stained with uranyl acetate and lead citrate. These sections were subsequently examined under a JEM-1400-FLASH transmission electron microscope for ultrastructural analysis.

### Tumor xenograft model

Four-week-old female BALB/c-nu mice, weighing 15–18 g were obtained from Vital River Company (Vital River, Guangdong, China). Mice were housed at the Animal Experimental Center of Guangxi Medical University. The mice were randomly assigned to the SKOV3, shNC, shRNA-1 or shRNA-2 group. All mice were administered a subcutaneous injection of 5 × 10^5^ cells suspended in 0.1 mL of medium devoid of FBS. The tumor became visible approximately 1-week postinoculation. Every 2 days, the minimum short diameter (W) and maximum long diameter (L) of the tumors were measured with a caliper. The tumor volume (V, in mm^3^) was determined using the formula: V = (L × W^2^)/2. Twenty-five days after implantation, the tumors were removed and photographed. The in vivo experimental procedures were approved by the Ethics Committee of Guangxi Medical University.

### Statistical analysis

Differential gene analysis was conducted using the GEPIA platform, available at http://gepia.cancer-pku.cn/. Survival curves were derived through the Kaplan–Meier method, accessible on the Kaplan–Meier plotter platform at http://kmplot.com/analysis/. KEGG pathway analysis was performed via https://www.kegg.jp/. The data were analyzed using GraphPad Prism and presented as the means ± SD. Comparisons between two samples were made using the *t*-test, while comparisons between multiple groups were performed via one-way ANOVA test. Significance was determined at a *P*-value < 0.05, with the following designations: not significant (ns, *P* > 0.05), *P < 0.05, **P < 0.01, and ***P < 0.001).

## Results

### ATP6V1B1 is highly expressed in ovarian neoplasia samples

**Fig. 1:**
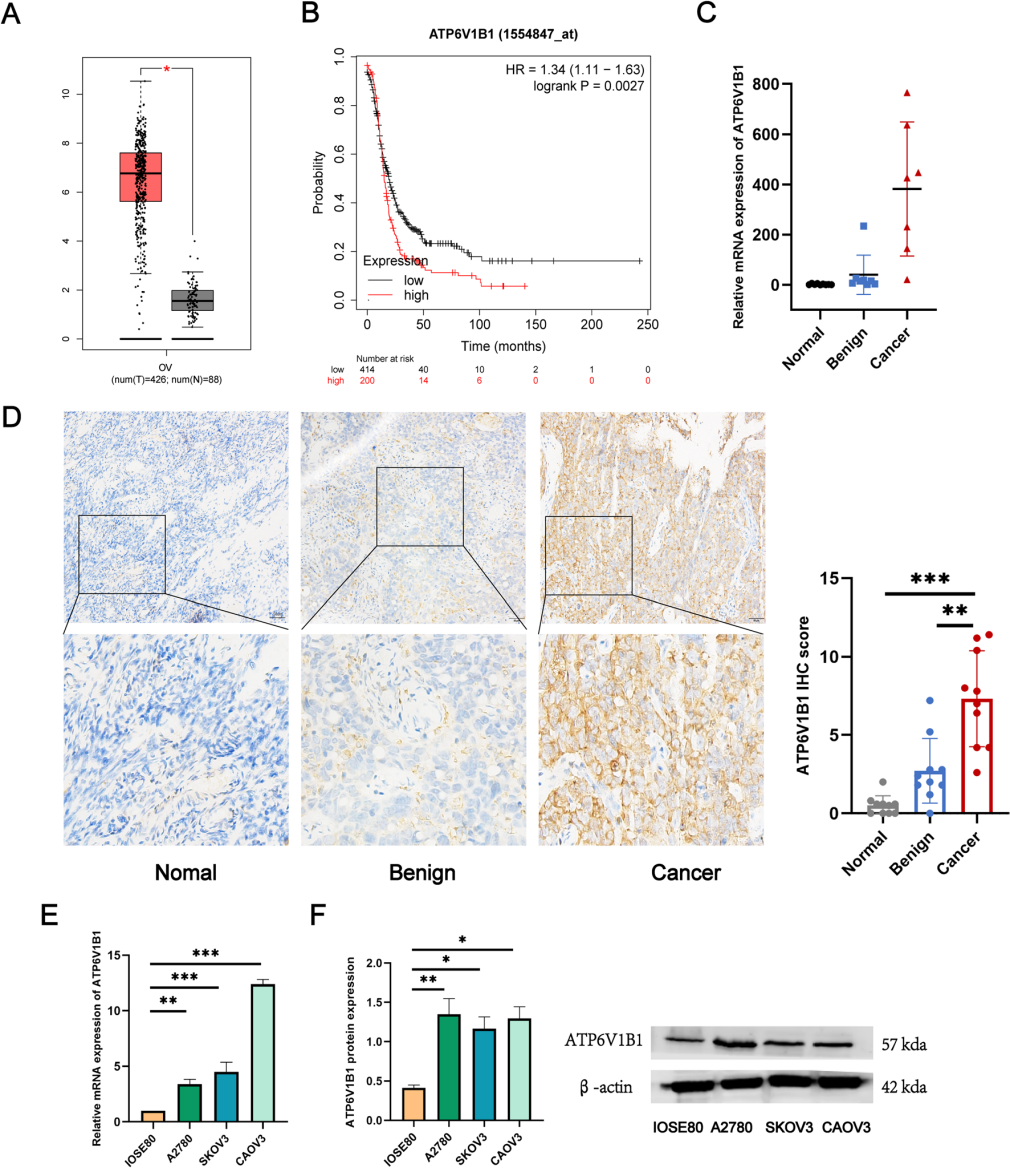
The expression of ATP6V1B1 and its correlation with the progression-free survival time of patients with ovarian cancer. **A** Boxplot of ATP6V1B1 in ovarian cancer. *TPM* transcripts per million. **B** Comparison of progression-free survival between patients with high and low ATP6V1B1 expression by Kaplan‒Meier plotter. **C** Relative mRNA expression of ATP6V1B1 in normal ovarian tissue (*n* = 9), benign tumor tissue (*n* = 8) and ovarian cancer tissue (*n* = 7). **D** IHC revealed ATP6V1B1 expression in normal ovarian tissue (*n* = 10), borderline tumor tissue (*n* = 10) and ovarian cancer tissue (*n* = 10); magnification, ×10, scale bar, 50 μm. **E**, **F** Relative mRNA and protein expression of ATP6V1B1 in the ovarian epithelial cell line IOSE80 and the ovarian cancer cell lines A2780, SKOV3, and CAOV3

As depicted in Fig. 1A, ATP6V1B1 levels were markedly elevated in ovarian cancer (OC) samples compared with nontumor. Furthermore, Kaplan‒Meier plotter analysis demonstrated that patients with higher ATP6V1B1 expression exhibited shorter progression-free survival (PFS) times than patients with lower ATP6V1B1 expression (Fig. 1B). Here, we examined the expression of ATP6V1B1 mRNA and protein in normal ovarian tissues, benign tumor tissues and OC tissues by RT‒qPCR and immunohistochemistry (IHC). Notably, ATP6V1B1 expression was greater in OC tissues than in normal or benign tumor tissues (Fig. 1C, D). We further compared ATP6V1B1 expression in normal ovarian cells (IOSE80) with that in ovarian cancer cell lines (A2780, SKOV3, CAOV3). Figure 1E, F show that, compared to IOSE80 cells, ovarian cancer cell lines exhibited significantly increased ATP6V1B1 transcript and protein levels, exhibiting a 2.9- to 3.4-fold increase (*P* < 0.05). The above results indicate that ATP6V1B1 is overexpressed in ovarian cancer.

### Effects of ATP6V1B1 on the proliferation of ovarian cancer cells

**Fig. 2:**
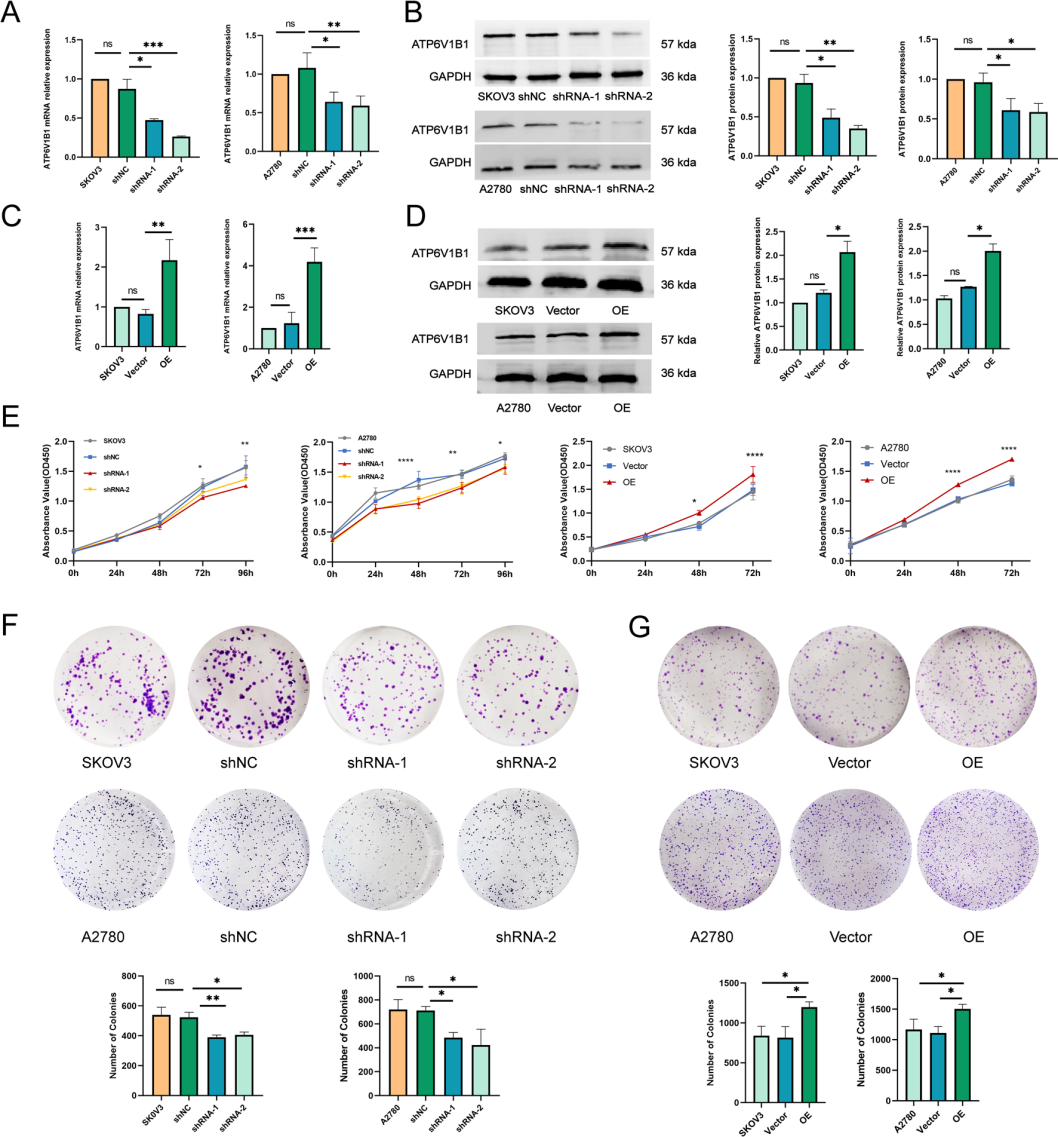
ATP6V1B1 affects ovarian cancer cell proliferation. The mRNA expression and protein expression levels of ATP6V1B1 after ATP6V1B1 silencing (**A**, **B**) and overexpression (**C**, **D**) in SKOV3 and A2780 cells were detected using RT‒qPCR and western blot respectively. Cell viability was determined by CCK-8 assays (**E**) and colony formation assays (**F**, **G**)

To investigate the role of ATP6V1B1 in ovarian cancer, we downregulated and upregulated ATP6V1B1 expression in SKOV3 and A2780 cells, respectively. The stable knockdown and overexpression efficiencies were verified by RT‒qPCR and Western blotting (Fig. 2A–D). Subsequently, we explored the impact of ATP6V1B1 on cancer cell growth and proliferation using these cell lines. CCK-8 assays demonstrated that silencing ATP6V1B1 inhibited the proliferation of both A2780 and SKOV3 cells. In contrast, the overexpression of ATP6V1B1 increased the proliferation of cells (Fig. 2E). Colony formation assays revealed a 23–26% decrease in colony formation in SKOV3 cells and a 32–41% reduction in colony formation in A2780 cells following ATP6V1B1 knockdown (Fig. 2F). In contrast, the overexpression of ATP6V1B1 increased the colony formation ability of the cells (Fig. 2G). In summary, ATP6V1B1 can promote the proliferation of ovarian cancer cells.

### Effects of ATP6V1B1 on the migration and invasion of ovarian cancer cells

**Fig. 3:**
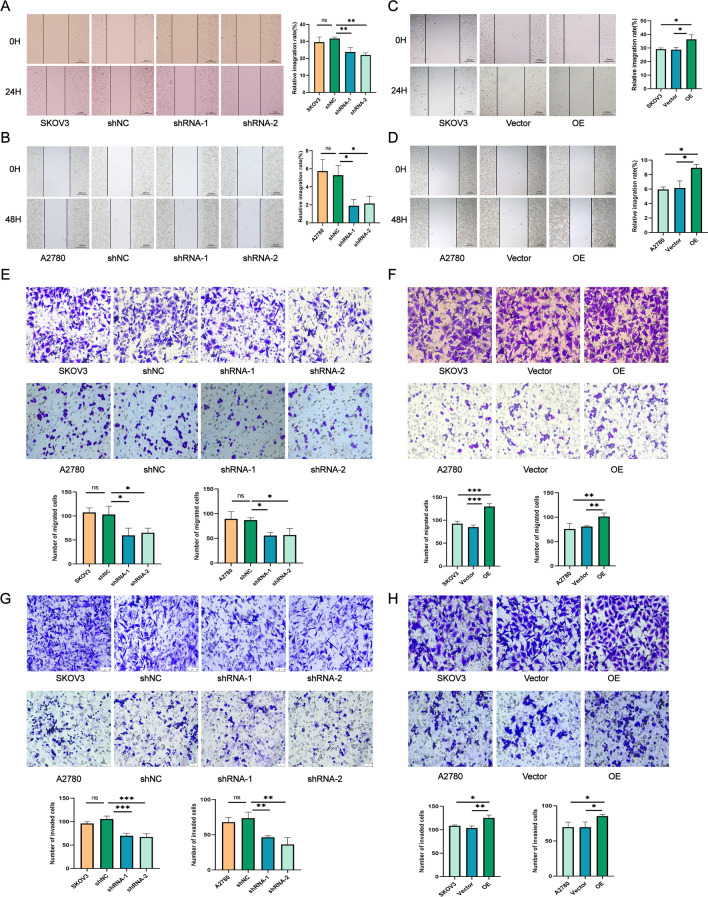
Interference with ATP6V1B1 affects ovarian cancer cell migration and invasion ability. Cell migration ability was assessed using the wound healing assay and Transwell assay (**A**–**F**). Cell invasion ability was detected using the Matrigel transwell assay (**G**, **H**)

V-ATPase has been demonstrated to promote tumor cell migration and invasion by enhancing MMP activity and actin interactions. [22]. In our research, we investigated whether ATP6V1B1 affects the migration and invasion of ovarian cancer cells. Therefore, scratch healing and transwell experiments were carried out. The wound-healing assay results showed that, compared with the control, ATP6V1B1 knockdown decreased the healing rate of the scratch area in SKOV3 (Fig. 3A) and A2780 (Fig. 3B) cells, while ATP6V1B1 overexpression increased the lateral migration capacity of the cells compared to that of the control group (Fig. 3C, D). Transwell assays revealed that the number of migrating cells was reduced in the ATP6V1B1 knockdown group, while the number of migrating cells was increased in the ATP6V1B1 overexpression group (Fig. 3E, F). Additionally, decreasing ATP6V1B1 levels substantially reduced cancer cell invasion through the Matrigel extracellular matrix in both cell lines (Fig. 3G). The invasion of ovarian cancer cells was enhanced by the overexpression of ATP6V1B1 (Fig. 3H). Differential expression was observed within 24 h in SKOV3 cells, whereas this difference was observed after 48 h in A2780 cells. These findings suggest that ATP6V1B1 knockdown hampers ovarian cancer cell migration and invasion.

### ATP6V1B1 affects the mTOR/autophagy pathway

**Fig. 4:**
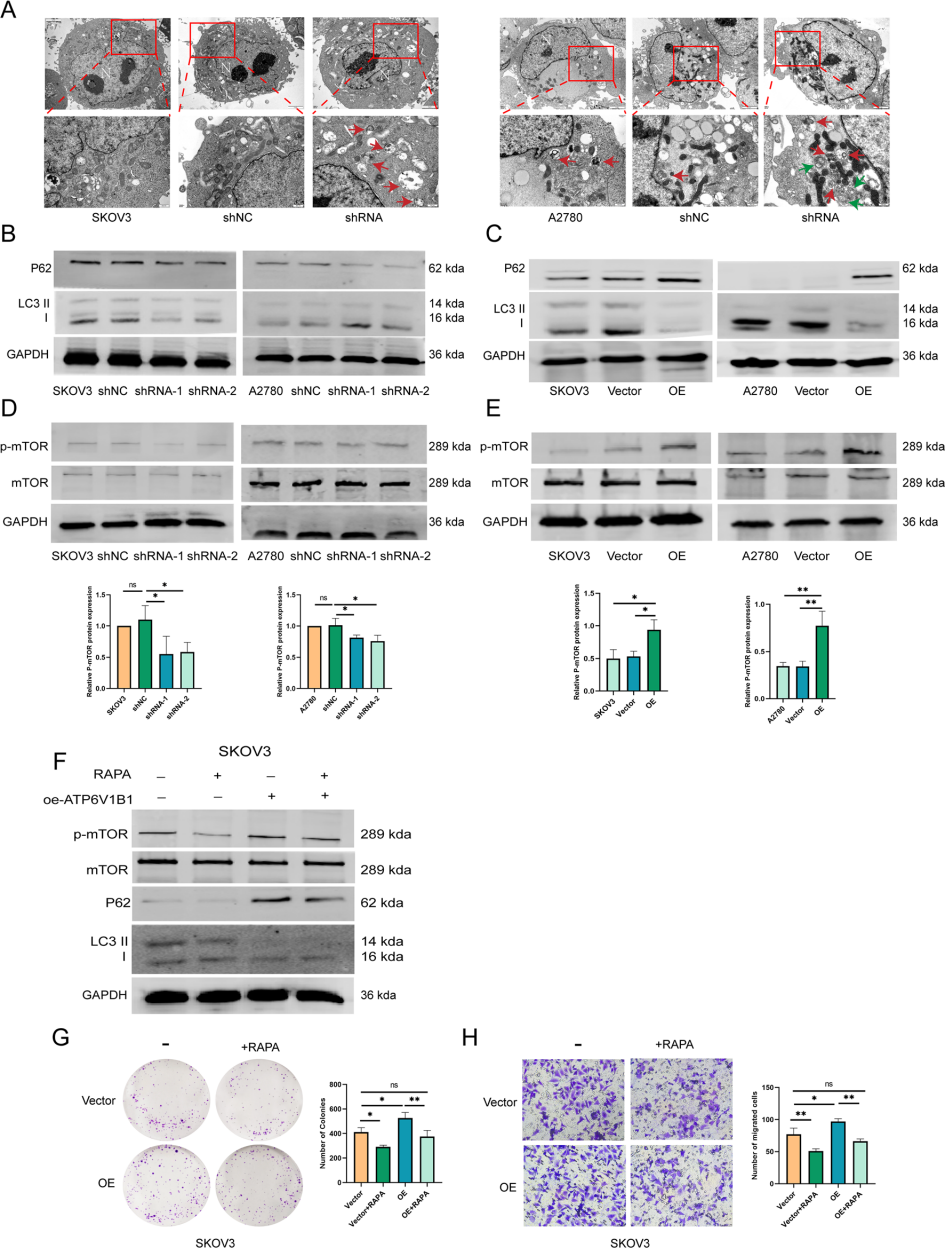
ATP6V1B1 is involved in the mTOR/autophagy signaling pathway. **A** Transmission electron microscopy (TEM) revealed the autophagic microstructure of SKOV3 and A2780 cells. Green arrows indicate a cellular autophagic microstructure with a double-layer structure, while the red arrow points to an autophagolysosome. Magnification ×8000, Scale bar, 2 μm. Magnification ×20,000, Scale bar, 500 nm. **B**, **C** The autophagy-related proteins LC3B and P62 were detected by Western blotting. **D**, **E** mTOR/p-mTOR protein expression was detected by Western blotting. **F** Western blotting analysis of the expression of p-mTOR, mTOR, P62 and LC3B in SKOV3 cells treated with an mTOR inhibitor (rapamycin, 1 µM inhibitor for 24 h). **G**, **H** Cell proliferation and Transwell migration were detected in SKOV3 cells treated with rapamycin

Using the Kyoto Encyclopedia of Genes and Genomes database (KEGG), we found that ATP6V1B1 is involved in the mTOR signaling pathway and regulates autophagy. V-ATPase regulates mTOR activity, affecting autophagy [9]. The involvement of ATP6V1B1 in regulating the cell cycle of ovarian cancer has been established [21]. In this study, our focus was on determining how ATP6V1B1 influences the cellular death process. TEM revealed no significant changes in the morphology of the control cells and few autophagosomes were present. In contrast, cells in the shRNA group had swollen rough endoplasmic reticula and mitochondria and an increased number of autophagosomes (Fig. 4A). We used Western blotting to determine the expression of LC3B and P62, which are important proteins in the autophagy pathway. LC3II/I expression was increased and P62 expression was decreased in the ATP6V1B1 knockdown group (Fig. 4B); conversely, LC3II/I expression was decreased and P62 expression was increased in the ATP6V1B1 overexpression group (Fig. 4C). These results indicated that ATP6V1B1 knockdown induces autophagy at the protein level in ovarian cancer cells. The V1 domain of V-ATPase interacts with Rheb and the Ragulator complex, which affects the activation of mTORC1 [23]. Our results showed a decrease in the levels of phosphorylated mTOR (p-mTOR) in the ATP6V1B1 knockdown group (Fig. 4D), but an increase in the levels of p-mTOR in the ATP6V1B1 overexpression group (Fig. 4E). The overexpression of ATP6V1B1 promoted the expression of p-mTOR and P62 in SKOV3 cells, and this effect was abolished by the administration of the mTOR inhibitor rapamycin (RAPA) (Fig. 4F). The results of the colony formation and Transwell assays also indicated that the promotion of cell proliferation and migration by overexpression of ATP6V1B1 was lost when the rapamycin was used (Fig. 4G, H). Those results suggest that ATP6V1B1 triggers mTOR protein expression in ovarian cancer cells and blocks autophagy.

### Downregulation of ATP6V1B1 enhances the sensitivity of ovarian cancer cells

**Fig. 5:**
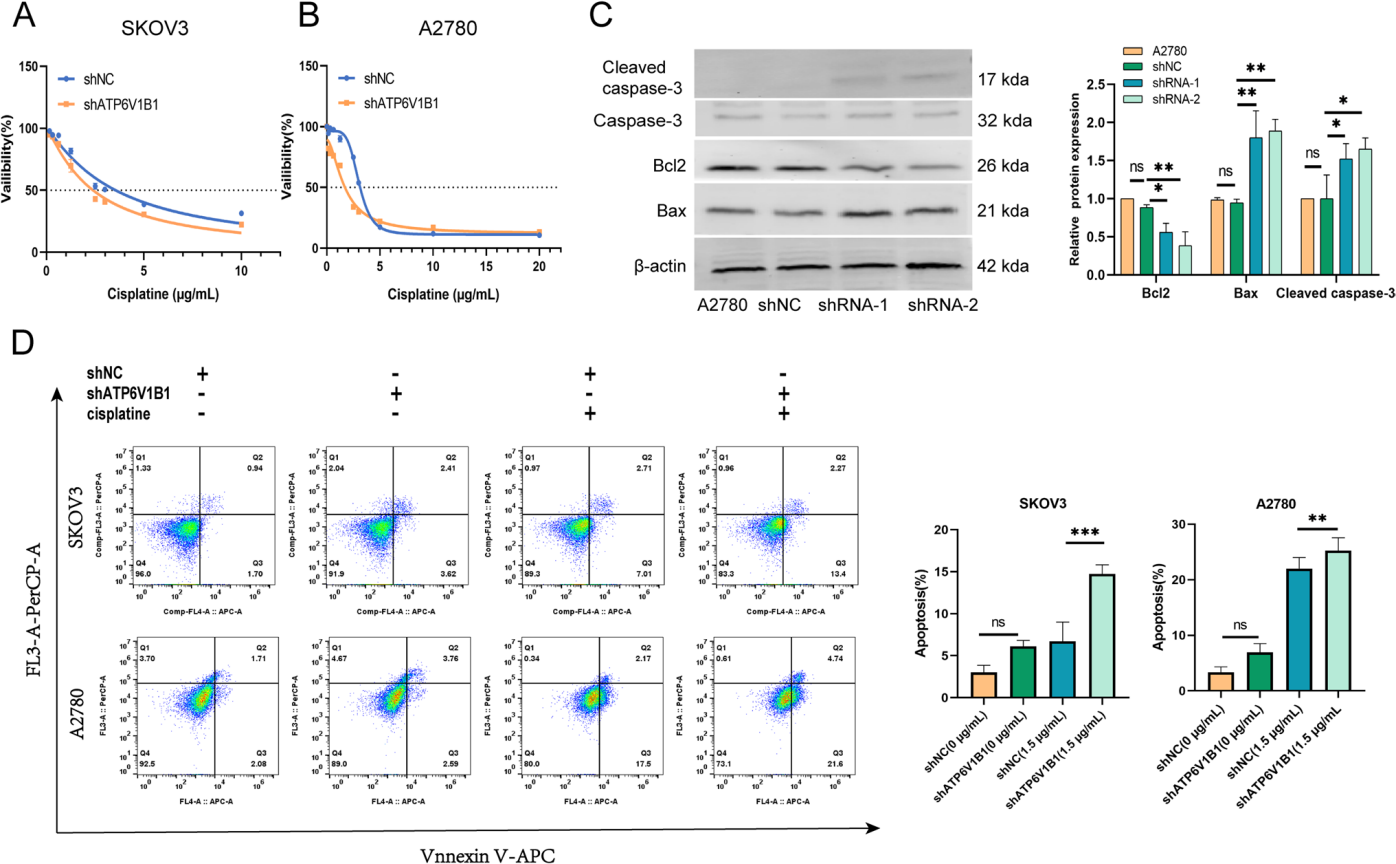
ATP6V1B1 silencing enhances cisplatin sensitivity in ovarian cancer cells. **A**, **B** SKOV3 and A2780 cells were incubated with a various concentration of cisplatin (0, 0.08, 0.16, 0.31, 0.63, 1.25, 2.50, 3.00, 5.00, 10.00, or 20.00 µg/mL) for 24 h. CCK8 assays were performed to determine the IC_50_ of cisplatin in SKOV3 (shNC IC_50_ = 3.55 µg/mL, sh-ATP6V1B1 IC_50_ = 2.39 µg/mL) and A2780 (shNC IC_50_ = 3.03 µg/mL, sh-ATP6V1B1 IC_50_ = 1.68 µg/mL) cells. **C** The protein expression of caspase-3, cleaved caspase-3, Bax and Bcl-2 was detected by Western blotting. **D** The apoptosis of ovarian cancer cells exposed to 1.5 µg/mL cisplatin for 24 h was detected by flow cytometry

Tumor cells increase extracellular acidification by upregulating the expression of V-ATPase, which induces drug protonation and reduces the ability of the drug to enter the cells [24]. Han et al. [21] demonstrated high ATP6V1B1 expression in platinum-resistant ovarian cancer tissues. Our study investigated the potential impact of ATP6V1B1 on cell viability and platinum sensitivity. Using the CCK8 assay, we evaluated the IC_50_ of cisplatin in SKOV3 and A2780 cells after ATP6V1B1 attenuation. Our results revealed a decrease in the IC_50_ from 3.55 to 2.39 µg/mL in SKOV3 cells, and a reduction in IC_50_ from 3.03 to 1.68 µg/mL in A2780 cells (Fig. 5A, B). After knocking down ATP6V1B1 in A2780 cells, there was a noteworthy reduction in the expression of Bcl2. Concurrently, an increase in the levels of Bax and cleaved caspase-3 was observed (Fig. 5C). Cell apoptosis was detected by flow cytometry using V-APC/AAD double staining. Compared with cisplatin treatment alone, ATP6V1B1 inhibition and cisplatin treatment synergistically increased the percentage of apoptotic ovarian cancer cells (Fig. 5D).

### Downregulation of ATP6V1B1 inhibits the growth of ovarian tumors in vivo

**Fig. 6:**
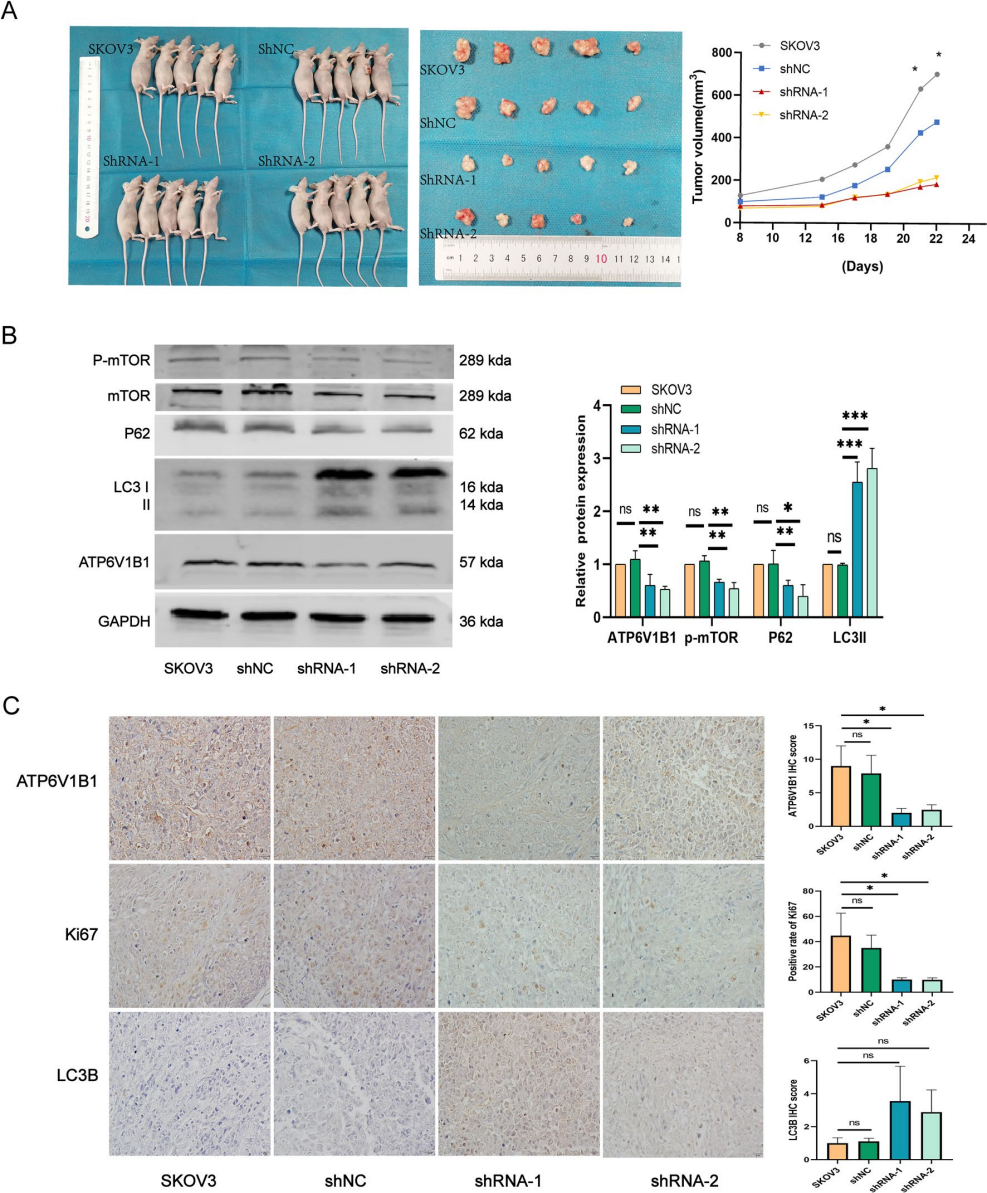
Interference with ATP6V1B1 inhibits the growth of ovarian cancer xenografts. **A** Xenograft tumor models were generated with SKOV3 cells. Tumor volumes were measured regularly, and the data points are the mean tumor volumes ± SDs. **B** The relative protein expression levels of ATP6V1B1, mTOR, phosphorylated mTOR, LC3B, P62, and GAPDH in xenograft tumors were detected via Western blotting. **C** Immunohistochemical staining results for ATP6V1B1, LC3B and Ki67 in ovarian xenograft tumors; magnification, ×40, scale bar, 20 μm

To assess the role of ATP6V1B1 in ovarian cancer progression in vivo, we established a nude mouse tumor xenograft model using the SKOV3 cell line. Our results showed that the average volume of transplanted tumors was reduced in the ATP6V1B1 knockdown group (Fig. 6A). We validated the expression of ATP6V1B1 and further demonstrated the link between ATP6V1B1 and the mTOR/autophagy pathway. The expression levels of ATP6V1B1 and P62 were lower in the ATP6V1B1 knockdown group, whereas the expression of the LC3II protein was increased (Fig. 6B). The immunohistochemistry results also showed that the protein expression of ATP6V1B1 and Ki67 was reduced, while the protein expression of LC3B was increased in the ATP6V1B1 knockout group. However, LC3B expression may be highly variable among transplants and the increase in LC3B expression was not statistically significant (Fig. 6C). These results suggest that disruption of ATP6V1B1 may induce lethal autophagy by inhibiting mTOR, thereby suppressing ovarian cancer tumor growth in vivo.

## Discussion

In this study, we investigated the role of ATP6V1B1 in the pathogenesis of ovarian cancer. We observed the overexpression of ATP6V1B1 in ovarian cancer tissues and cells, and silencing ATP6V1B1 suppressed the proliferation, migration, and invasion abilities of ovarian cancer cells, while increasing the sensitivity of cells to cisplatin. We also observed that the silencing of ATP6V1B1 promoted autophagy by inhibiting the activation of mTOR.

The role of V-ATPase in tumorigenesis is well-established [9, 13]. However, more research is needed to elucidate the role of the different subunits of the V-ATPase in cancer development to clarify its reliability as a biomarker and its therapeutic significance. Analysis of the GEPIA database revealed that ATP6V1B1 expression was significantly increased in ovarian cancer tissue, and Kaplan‒Meier plotter revealed that ATP6V1B1 expression was associated with poor progression-free survival. We observed overexpression of ATP6V1B1 in ovarian cancer tissues and three distinct ovarian cancer cell lines. This finding was consistent with previous research demonstrating the overexpression of ATP6V1B1 in non serous ovarian cancer cells [21]. Interestingly, our study revealed inconsistent expression patterns of ATP6V1B1 mRNA and protein in CAOV3 cells. This stochastic behavior indicates that the protein expression of ATP6V1B1 exhibits random fluctuations in cells, in line with the concept of gene expression noise [25]. High levels of mRNA can suppress protein translation, leading to minimal protein expression [26]. The differential expression of ATP6V1B1 at the transcriptional and translational levels in cells warrants further investigation to elucidate its specific mechanisms. We also showed that reduced ATP6V1B1 expression inhibited the proliferation, migration and invasion of ovarian cancer cells. Ovarian cancer cells may promote cellular migration and invasion by upregulating ATP6V1B1 to enhance MMP activity or interaction with actin [8, 27], but further mechanistic studies will be needed. In summary, our study further confirmed the oncogenic role of ATP6V1B1 in ovarian cancer.

KEGG pathway enrichment analysis suggested that ATP6V1B1 may be involved in the mTOR/autophagy signaling pathway. The PI3K/AKT/mTOR pathway is a classical signaling pathway activated by insulin stimulation. However, amino acids can also directly activate mTORC1 activity via the lysosome [28]. Under amino acids stimulation, the Ragulator complex interacts with Rheb on the surface of lysosomes; as a result, mTORC1 is recruited to the lysosomal membrane and activated. mTORC1 mediates the activation of S6K1 and 4E-BP1, which promotes mRNA maturation and protein translation [29]. In our study, we observed that ATP6V1B1 knockdown decreased the expression of phosphorylated mTOR. Conversely, the overexpression of ATP6V1B1 led to an increase in the level of phosphorylated mTOR. The overexpression of ATP6V1B1 induced mTOR activation and increased cell proliferation and migration. This effect could be blocked by the mTOR inhibitor RAPA.

Autophagy is known to have dual roles in cancer. Numerous studies have indicated that autophagy facilitates tumor cell growth, and many clinical interventions, such as the drugs CQ and HCQ, which are currently used to inhibit autophagy, focus on inhibiting autophagy [30]. However, other studies have suggested that inhibiting autophagy in cancer therapy is not good, as autophagy facilitates the ability of the immune system to identify immunogenic tumor cells and initiate the immune response [31–33]. When there is a lack of energy, mTORC1 reduces the phosphorylation of ULK1 at Ser757 and promotes autophagy [29, 30]. Inhibition of the mTOR signaling pathway in tumor cells leads to apoptosis and lethal autophagy [34, 35]. Although studies have shown that ATPases regulate autophagy, whether ATP6V1B1 regulates autophagy in tumor cells remains unknown. We observed that, after transfection with ATP6V1B1, the expression of P62 decreased, while the expression of the autophagy-related protein LC3II increased in ovarian cancer cells. Conversely, ATP6V1B1 overexpression resulted in elevated P62 protein expression and a reduced LC3II expression. Our results suggest that inhibition of ATP6V1B1 can induce lethal autophagy in tumor cells, thereby inhibiting cell growth. These results are consistent with the study by Yuhong Liu, who showed that gefitinib combined with rapamycin treatment inhibited the proliferation of lung cancer cells by upregulating autophagy [36]. Kulshrestha’s research revealed that ATP6V0a2 promotes chemoresistance in ovarian cancer cells through the induction of autophagy [37]. There are three types of autophagy: macroautophagy, microautophagy and chaperone-mediated autophagy. Macroautophagy is the quintessential autophagy pathway and plays a dominant role in regulating the physiological activities of organisms. It encapsulates cellular metabolites such as damaged organelles or misfolded proteins in double-membrane vesicles, which are then transported to lysosomes for degradation. Microautophagy involves direct phagocytosis and degradation of cellular metabolites by lysosomal membranes through invagination. Chaperone-mediated autophagy requires the HSC70 complex to recognize proteins with KFERQ-like sequences and then transport them to the lysosome for degradation [14]. V-ATPase is essential for macroautophagy due to the requirement for the fusion of autophagosomes with lysosomes. V-ATPase enzymes are responsible for maintaining the acidic environment within lysosomes, which is conducive to the activity of various proteases and hydrolases. In contrast, microautophagy does not involve direct contact between membranes, suggesting that V-ATPase may not be indispensable in this context [38, 39]. Our study revealed that ATP6V1B1 may play an important role in the process of macroautophagy in ovarian cancer cells, but more specific interaction experiments are needed to demonstrate the role of ATP6V1B1 in this process.

Despite advancements in drug research for ovarian cancer treatment, chemotherapy resistance continues to hinder efforts to extend the survival of ovarian cancer patients. Cellular epigenetic modifications, apoptosis dysregulation, reduced drug transport, and drug target mutations are potential factors leading to drug resistance in tumor cells [40, 41]. Normal cells have an acidic intracellular pH and a neutral extracellular pH, which favors the uptake of basic drugs. However, tumor cells increase the expression of V-ATPase to resist alkaline drug uptake. Inhibition of V-ATPase inhibits the proliferation of drug-resistant ovarian cancer cells and enhances their sensitivity to trastuzumab [42, 43]. Our findings indicate that ATP6V1B1 knockdown increases the cisplatin sensitivity of ovarian cancer cells. This process may be affected by the acidic intracellular environment or by cellular autophagy.

In summary, our study indicated that ATP6V1B1 is an oncogene involved in the pathogenesis of ovarian cancer. The suppression of ATP6V1B1 expression can inhibit mTOR activation, thereby inducing lethal autophagy, which in turn inhibits cell growth and enhances sensitivity to cisplatin.

## Conclusion

This study revealed that ATP6V1B1 is an oncogenic factor that is highly expressed in ovarian cancer tissues and cell lines. The overexpression of ATP6V1B1 contributes to a malignant ovarian phenotype and cisplatin chemoresistance via the mTOR/autophagy pathway. These findings suggest that ATP6V1B1 may be a potential biomarker for cancer prognosis and a therapeutic target against cisplatin chemoresistance in ovarian cancer.

## Supplementary Information

Below is the link to the electronic supplementary material.

Supplementary file1 (PDF 276 KB)

## Data Availability

The raw data supporting the conclusions of this article will be made available by the authors, without undue reservation.

## References

[1] Armstrong DK et al (2022) NCCN Guidelines^®^ Insights: ovarian cancer, version 3.2022. J Natl Compr Canc Netw 20:972–980. 10.6004/jnccn.2022.0047 36075393 10.6004/jnccn.2022.0047

[2] Tompkins JD, Wu X, Her C (2012) MutS homologue hMSH5: role in cisplatin-induced DNA damage response. Mol Cancer 11:10. 10.1186/1476-4598-11-10 22401567 10.1186/1476-4598-11-10 PMC3325843

[3] Mirza MR et al (2020) The forefront of ovarian cancer therapy: update on PARP inhibitors. Ann Oncol 31:1148–1159. 10.1016/j.annonc.2020.06.004 32569725 10.1016/j.annonc.2020.06.004

[4] Pamarthy S, Kulshrestha A, Katara GK, Beaman KD (2018) The curious case of vacuolar ATPase: regulation of signaling pathways. Mol Cancer 17:41. 10.1186/s12943-018-0811-3 29448933 10.1186/s12943-018-0811-3 PMC5815226

[5] Couto-Vieira J et al (2020) Multi-cancer V-ATPase molecular signatures: a distinctive balance of subunit C isoforms in esophageal carcinoma. EBioMedicine 51:102581. 10.1016/j.ebiom.2019.11.042 31901859 10.1016/j.ebiom.2019.11.042 PMC6948166

[6] Lee YY et al (2015) Proton pump inhibitors enhance the effects of cytotoxic agents in chemoresistant epithelial ovarian carcinoma. Oncotarget 6:35040–35050. 10.18632/oncotarget.5319 26418900 10.18632/oncotarget.5319 PMC4741507

[7] Nishie M et al (2021) Downregulated ATP6V1B1 expression acidifies the intracellular environment of cancer cells leading to resistance to antibody-dependent cellular cytotoxicity. Cancer Immunol Immunother 70:817–830. 10.1007/s00262-020-02732-3 33000417 10.1007/s00262-020-02732-3 PMC10991185

[8] Licon-Munoz Y, Michel V, Fordyce CA, Parra KJ (2017) F-actin reorganization by V-ATPase inhibition in prostate cancer. Biol Open 6:1734–1744. 10.1242/bio.028837 29038303 10.1242/bio.028837 PMC5703614

[9] Perez-Sayans M et al (2010) Measurement of ATP6V1C1 expression in brush cytology samples as a diagnostic and prognostic marker in oral squamous cell carcinoma. Cancer Biol Ther 9:1057–1064. 10.4161/cbt.9.12.11880 20404513 10.4161/cbt.9.12.11880

[10] Liu P, Chen H, Han L, Zou X, Shen W (2015) Expression and role of V1A subunit of V-ATPases in gastric cancer cells. Int J Clin Oncol 20:725–735. 10.1007/s10147-015-0782-y 25652905 10.1007/s10147-015-0782-y

[11] Damaghi M, Wojtkowiak JW, Gillies RJ (2013) pH sensing and regulation in cancer. Front Physiol 4:370. 10.3389/fphys.2013.00370 24381558 10.3389/fphys.2013.00370 PMC3865727

[12] Cotter K, Stransky L, McGuire C, Forgac M (2015) Recent insights into the structure, regulation, and function of the V-ATPases. Trends Biochem Sci 40:611–622. 10.1016/j.tibs.2015.08.005 26410601 10.1016/j.tibs.2015.08.005 PMC4589219

[13] Kulshrestha A et al (2015) Vacuolar ATPase ‘a2’ isoform exhibits distinct cell surface accumulation and modulates matrix metalloproteinase activity in ovarian cancer. Oncotarget 6:3797–3810. 10.18632/oncotarget.2902 25686833 10.18632/oncotarget.2902 PMC4414154

[14] Mizushima N, Levine B (2020) Autophagy in human diseases. N Engl J Med 383:1564–1576. 10.1056/NEJMra2022774 33053285 10.1056/NEJMra2022774

[15] Anding AL, Baehrecke EH (2017) Cleaning house: selective autophagy of organelles. Dev Cell 41:10–22. 10.1016/j.devcel.2017.02.016 28399394 10.1016/j.devcel.2017.02.016 PMC5395098

[16] Dikic I, Elazar Z (2018) Mechanism and medical implications of mammalian autophagy. Nat Rev Mol Cell Biol 19:349–364. 10.1038/s41580-018-0003-4 29618831 10.1038/s41580-018-0003-4

[17] Mathew R et al (2009) Autophagy suppresses tumorigenesis through elimination of p62. Cell 137:1062–1075. 10.1016/j.cell.2009.03.048 19524509 10.1016/j.cell.2009.03.048 PMC2802318

[18] Chen F, Kang R, Liu J, Tang D (2022) The V-ATPases in cancer and cell death. Cancer Gene Ther. 10.1038/s41417-022-00477-y 35504950 10.1038/s41417-022-00477-y PMC9063253

[19] Mohammad RM et al (2015) Broad targeting of resistance to apoptosis in cancer. Semin Cancer Biol 35(Suppl):S78–S103. 10.1016/j.semcancer.2015.03.001 25936818 10.1016/j.semcancer.2015.03.001 PMC4720504

[20] Yao X et al (2021) The ATPase subunit of ATP6V1C1 inhibits autophagy and enhances radiotherapy resistance in esophageal squamous cell carcinoma. Gene 768:145261. 10.1016/j.gene.2020.145261 33183740 10.1016/j.gene.2020.145261

[21] Han GH, Yun H, Chung JY, Kim JH, Cho H (2023) High ATP6V1B1 expression is associated with poor prognosis and platinum-based chemotherapy resistance in epithelial ovarian cancer. Oncol Rep 49:102. 10.3892/or.2023.8539 36999629 10.3892/or.2023.8539 PMC10091082

[22] Flinck M et al (2020) The vacuolar H(+) ATPase α3 subunit negatively regulates migration and invasion of human pancreatic ductal adenocarcinoma cells. Cells 9:465. 10.3390/cells9020465 32085585 10.3390/cells9020465 PMC7072798

[23] Zoncu R et al (2011) mTORC1 senses lysosomal amino acids through an inside-out mechanism that requires the vacuolar H(+)-ATPase. Science 334:678–683. 10.1126/science.1207056 22053050 10.1126/science.1207056 PMC3211112

[24] Hindenburg AA et al (1989) Intracellular distribution and pharmacokinetics of daunorubicin in anthracycline-sensitive and -resistant HL-60 cells. Cancer Res 49:4607–4614 2545346

[25] Singh A (2011) Negative feedback through mRNA provides the best control of gene-expression noise. IEEE Trans Nanobioscience 10:194–200. 10.1109/tnb.2011.2168826 22020106 10.1109/TNB.2011.2168826

[26] Weidemann DE, Holehouse J, Singh A, Grima R, Hauf S (2023) The minimal intrinsic stochasticity of constitutively expressed eukaryotic genes is sub-Poissonian. Sci Adv 9:eadh5138. 10.1126/sciadv.adh5138 37556551 10.1126/sciadv.adh5138 PMC10411910

[27] Chung C et al (2011) The vacuolar-ATPase modulates matrix metalloproteinase isoforms in human pancreatic cancer. Lab Invest 91:732–743. 10.1038/labinvest.2011.8 21339745 10.1038/labinvest.2011.8 PMC3084324

[28] Stransky LA, Forgac M (2015) Amino acid availability modulates vacuolar H^+^-ATPase assembly. J Biol Chem 290:27360–27369. 10.1074/jbc.M115.659128 26378229 10.1074/jbc.M115.659128 PMC4646367

[29] Sancak Y et al (2010) Ragulator-Rag complex targets mTORC1 to the lysosomal surface and is necessary for its activation by amino acids. Cell 141:290–303. 10.1016/j.cell.2010.02.024 20381137 10.1016/j.cell.2010.02.024 PMC3024592

[30] Levy JMM, Towers CG, Thorburn A (2017) Targeting autophagy in cancer. Nat Rev Cancer 17:528–542. 10.1038/nrc.2017.53 28751651 10.1038/nrc.2017.53 PMC5975367

[31] Gao L, Chen Y (2021) Autophagy controls programmed death-ligand 1 expression on cancer cells (Review). Biomed Rep 15:84. 10.3892/br.2021.1460 34512972 10.3892/br.2021.1460 PMC8411486

[32] Huang J et al (2019) Effect of autophagy on expression of neutrophil programmed death ligand-1 in mice with sepsis. Zhonghua Wei Zhong Bing Ji Jiu Yi Xue 31:1091–1096. 10.3760/cma.j.issn.2095-4352.2019.09.007 31657331 10.3760/cma.j.issn.2095-4352.2019.09.007

[33] Booth L, Roberts JL, Poklepovic A, Dent P (2017) [pemetrexed + sildenafil], via autophagy-dependent HDAC downregulation, enhances the immunotherapy response of NSCLC cells. Cancer Biol Ther 18:705–714. 10.1080/15384047.2017.1362511 28812434 10.1080/15384047.2017.1362511 PMC5663410

[34] Singh J, Hussain Y, Meena A, Luqman S, Sinha RA (2023) Molecular regulation of autophagy and suppression of protein kinases by aescin, a triterpenoid saponin impedes lung cancer progression. Int J Biol Macromol 252:126328. 10.1016/j.ijbiomac.2023.126328 37579900 10.1016/j.ijbiomac.2023.126328

[35] Shi XZ et al (2023) Antitumor activity of berberine by activating autophagy and apoptosis in CAL-62 and BHT-101 anaplastic thyroid carcinoma cell lines. Drug Des Dev Ther 17:1889–1906. 10.2147/dddt.S406354 10.2147/DDDT.S406354 PMC10312214 37397788

[36] Liu Y et al (2022) Targeted co-delivery of gefitinib and rapamycin by aptamer-modified nanoparticles overcomes EGFR-TKI resistance in NSCLC via promoting autophagy. Int J Mol Sci 23:8025. 10.3390/ijms23148025 35887373 10.3390/ijms23148025 PMC9316473

[37] Kulshrestha A et al (2019) Targeting V-ATPase isoform restores cisplatin activity in resistant ovarian cancer: inhibition of autophagy, endosome function, and ERK/MEK pathway. J Oncol 2019:2343876. 10.1155/2019/2343876 31057611 10.1155/2019/2343876 PMC6463777

[38] Kanki T, Klionsky DJ (2008) Mitophagy in yeast occurs through a selective mechanism. J Biol Chem 283:32386–32393. 10.1074/jbc.M802403200 18818209 10.1074/jbc.M802403200 PMC2583303

[39] Kissová I et al (2007) Selective and non-selective autophagic degradation of mitochondria in yeast. Autophagy 3:329–336. 10.4161/auto.4034 17377488 10.4161/auto.4034

[40] Kuang Y et al (2021) Inhibition of microRNA let-7b expression by KDM2B promotes cancer progression by targeting EZH2 in ovarian cancer. Cancer Sci 112:231–242. 10.1111/cas.14708 33091189 10.1111/cas.14708 PMC7780014

[41] Kuang Y et al (2017) Histone demethylase KDM2B upregulates histone methyltransferase EZH2 expression and contributes to the progression of ovarian cancer in vitro and in vivo. Onco Targets Ther 10:3131–3144. 10.2147/ott.S134784 28706445 10.2147/OTT.S134784 PMC5495092

[42] Liao C, Hu B, Arno MJ, Panaretou B (2007) Genomic screening in vivo reveals the role played by vacuolar H^+^ ATPase and cytosolic acidification in sensitivity to DNA-damaging agents such as cisplatin. Mol Pharmacol 71:416–425. 10.1124/mol.106.030494 17093137 10.1124/mol.106.030494

[43] Marquardt D, Center MS (1991) Involvement of vacuolar H(+)-adenosine triphosphatase activity in multidrug resistance in HL60 cells. J Natl Cancer Inst 83:1098–1102. 10.1093/jnci/83.15.1098 1831509 10.1093/jnci/83.15.1098

