# Supplementary material for: ATP6V1B1 regulates ovarian cancer progression and cisplatin sensitivity through the mTOR/autophagy pathway
Source: Mol Cell Biochem. 2024 May 12;480(2):1013–26. doi: 10.1007/s11010-024-05025-w (PMC11835902; doi:10.1007/s11010-024-05025-w)
Supplement: Supplementary file 1 — Supplementary file1 (PDF 276 KB) [file 11010_2024_5025_MOESM1_ESM.pdf]

广西医科大学第一附属医院
THE FIRST AFFILIATED HOSPITAL OF
GUANGXI MEDICAL UNIVERSITY

# FIRST AFFILIATED HOSPITAL of GUANGXI MEDICAL UNIVERSITY
# ETHICAL REVIEW COMMITTEE Approval Notice

**Approval Number:** 2024-E047-01
**Title:** ATP6V1B1 regulates ovarian cancer progression and cisplatin sensitivity through the mTOR/autophagy pathway
**Research Contents:** Investigating the expression of ATP6V1B1 in ovarian cancer and its regulatory mechanisms in the oncogenesis and progression of the disease, this study aims to elucidate the mechanisms underlying ovarian cancer metastasis and to address the issue of resistance to platinum-based chemotherapeutics. The specific research objectives are as follows: (1) Evaluate the expression levels of the ATP6V1B1 gene in ovarian tissue, and investigate its potential correlations with clinical staging and prognostic outcomes in patients.(2) Evaluate the expression of ATP6V1B1 in ovarian cancer cells.(3) Knockdown and overexpression of ATP6V1B1 in ovarian cancer cells using plasmid vectors.(4) Investigation of the impact of ATP6V1B1 silencing and overexpression on cell proliferation, migration, invasion, and cell cycle regulation in ovarian cancer cells.(5) Determine cell viability (or IC50) in cells with either silenced or overexpressed ATP6V1B1 when exposed to a range of treatment concentrations. (6) Flow cytometric analysis of apoptosis in cells treated with cisplatin alone or in combination following ATP6V1B1 silencing or overexpression. (7) Investigation of the effect of ATP6V1B1 expression on the mTOR signaling pathway. (8) Study of ATP6V1B1's impact on cellular autophagy. (9) Establishment of a subcutaneous xenograft tumor model in BALB/c-nu mice with ATP6V1B1 knockdown ovarian cancer cells
**Applicant:** Yan Kuang
**Application Department:** Department of Gynecology, First Affiliated Hospital of Guangxi Medical University
**Date of Application:** January 18, 2024
**Date of Approval:** January 19, 2024
**Conclusion:** This paper fully considered and protected the rights and interests of the study objects. It meets the criteria of Ethical Review Committee. The Medical Ethics

Committee of First Affiliated Hospital of Guangxi Medical University has approved the protocol.

Signature:

(Vice) Director of Ethical Review Committee

First Affiliated Hospital of Guangxi Medical University

Date: January 19, 2024
